# Motivation for patient engagement in patient safety: a multi-perspective, explorative survey

**DOI:** 10.1186/s12913-024-11495-x

**Published:** 2024-09-11

**Authors:** Caroline Raab, Nikoloz Gambashidze, Larissa Brust, Matthias Weigl, Amelie Koch

**Affiliations:** 1 Institute for Patient Safety (IfPS), University Hospital Bonn, Venusberg-Campus 1, 53127 Bonn, Germany; 2 Department of Psychology, University of Innsbruck, Innsbruck, Austria

**Keywords:** Patient engagement, Patient safety, Motivation, Stakeholder, Qualitative analysis, Survey, Healthcare

## Abstract

**Background and Objectives:**

Despite increasing calls for more patient engagement in patient safety, limited knowledge remains on what actually motivates key stakeholders in healthcare to promote patient engagement. We therefore set out to survey key stakeholders of patient engagement in patient safety (i.e., patients, healthcare professionals, and managers). We aimed to identify and explore stakeholder’s distinct and shared motives for patient engagement.

**Methods:**

A stepwise Delphi method was applied, utilizing semi-structured online interviews for determination of stakeholder motives for patient engagement in patient safety. In a subsequent online survey, statements were evaluated and identified. 34 subject-matter experts from all relevant stakeholder groups completed the online interviews and 33 the online survey. We used content analysis approaches for qualitative and descriptive analyses for quantitative measures. Further, we evaluated the consensus on distinct and shared motives across stakeholder groups.

**Results:**

Seven key motives for patient engagement in patient safety were identified. Major motives attributed to patients were: (1) *To improve experiences and care outcomes for oneself*, as well as (2) *for future patients*, (3) *to express gratitude and appreciation*, (4) *to cope successfully with treatment-related emotions*. A motive shared by patients and professionals was (5) *to contribute actively to improved delivery of healthcare*. *To optimize patient safety*,* costs*,* and care processes* (6) was shared by professionals and managers. Lastly, (7) *to improve patient-provider relationships* was jointly shared by all stakeholder groups. For four motives (1, 2, 6, 7) consensus was established.

**Conclusions:**

In order to unlock the full potential of future interventions in patient engagement, a deeper understanding of stakeholder motives is essential. We identified a set of distinct and shared motives for patient engagement across relevant stakeholder groups. Our findings may inform future interventions in patient engagement that take account of the motivational foundations and aspirations of all stakeholders who are key for the success for collaborative patient safety and care improvements.

**Trial registration:**

ID DRKS00031837 (Date May 8, 2023).

## Background

According to the WHO, a key strategy in improving patient safety — defined as a framework that consistently lowers risks, reduces avoidable harm, and mitigates the impact of errors — is to actively engage patients and their families as the “coproducers of health” [1]. Patient engagement (PE) has been proposed as one of the “most powerful tool[s] to improve patient safety” [2]. Although definitions differ considerably, patient engagement can be defined as “patients, families, their representatives, and health professionals working in active partnership at various levels across the health care system — direct care, organizational design and governance, and policy making — to improve health and health care” [3]. Specifically for patient safety, this approach is based on the assumption that supporting safer care requires a collaborative effort among all relevant actors and stakeholders to mitigate risks and harm, as well as to promote safety [4–6]. Nonetheless, adoption of PE activities is low in the majority of healthcare systems, what has been attributed to various cultural, structural, and procedural barriers to effective implementation [7]. Mostly mentioned barriers in literature comprise lack of trust, communication barriers, time constraints, poor organizational culture, and lack of resources [6–9].

Whilst the study of barriers and facilitators to PE in healthcare institutions is attracting increasing interest, limited knowledge remains on stakeholders’ motives to engage in patient safety activities. Motivation is commonly defined as the driving force behind human actions and the process that initiates, directs, and maintains goal-oriented behaviors [10, 11]. Goals that adhere with individual needs enhance intensity and persistence of pursuit with, ultimately, better performance [12].

Previous investigations pointed to several motives for patients’ willingness to participate in healthcare [13, 14]. Alike, patients may also have reservations or report unwillingness to engage in patient safety measures [6]. Moreover, institution’s may be unwilling to involve patients [7] or consider patients’ input [15]. However, the construct of motivation has not yet been systematically examined so far in the context of PE in patient safety. In this study, we will focus on contents or the “what” of relevant stakeholder motives as a key precondition to effective patient engagement for promoting patient safety in care.

The current literature base on PE surveyed largely quality and healthcare improvement and to a lesser extent motivational aspects in patient safety; with the majority of available studies surveyed solely patients and their families. A study by McCarron, Noseworthy [16], for instance, has shown, that patients’ and their family’s motives to engage in PE depend on conditional circumstances and range from self-fulfillment, improving healthcare and learning new insights, expanding influence, as well as obtaining compensation or certain other perks. Mostly, patients and their representatives act in response to individual events, “often to give some meaning to tragedy by sharing their experience and expertise to raise awareness and catalyze change” [15, 17]. From the perspective of care professionals and providers, previous studies have shown that PE can improve the work life of clinicians and staff [18]. PE interventions can further improve the quality of care [19], decrease healthcare costs [18] and enhance the efficiency of healthcare systems [20]. Altogether, since available research on PE motives was inconsistent with regard to stakeholders, care sectors, and national contexts, and as the field of PE research is currently developing dynamically, we decided to explore motives of different stakeholders to obtain a comprehensive picture.

Beyond stakeholders’ individual motives, a gap in the knowledge base exists to what motives for PE in patient safety improvement are shared across stakeholder groups. The available literature does not provide insights on the consistency of motives across different groups of stakeholders. According to the Social Identity Approach to Motivation, if motives are shared among stakeholders, this fosters a sense of social connection and eventually contributes to reaching collective goals and may promote PE activities [21]. Therefore, we set out to generate new insights about shared motives between stakeholder groups for PE in patient safety.

## Research question

To explore stakeholder motives for PE in patient safety, we surveyed key stakeholder groups in healthcare, i.e., patients, healthcare professionals, and managers. Specifically, we sought to answer the following question: What unique and shared motives for PE in patient safety do different stakeholder groups report?

## Methods

### Research design and procedure

Our exploratory study used a Delphi-method approach drawing upon a step-wise aggregation of qualitative and quantitative analyses. This systematic approach is well-suited for consolidation of statements among a heterogeneous panel of interviewees with potentially divergent opinions. Previously, this method has been successfully applied to PE surveys [22]. All interview data was collected as part of a larger research project (Title: PEPS II - Barriers and facilitators of patient engagement activities to improve patient safety in healthcare facilities: A Delphi-based expert survey; registered in German Clinical Trials Register, ID DRKS00031837; registered May 8 2023). Whereas the main study aimed to investigate contextual and process factors for patient engagement in patient safety activities, this particular study was merely interested in stakeholder motives. Positive ethics approval was obtained prior to the study (Ethics Committee of Medical Faculty, Bonn University, 091/23-EP). Reporting of the results adheres to the COREQ Checklist for qualitative studies [23] as well as to recommendations for reporting Delphi studies [24].

### Participants

A convenience sample was established. Panelists were recruited through multiple methods, including email invitations using a snowball sampling approach. We sought to include panelists from key healthcare stakeholder groups relevant to PE activities, i.e., patients, healthcare professionals, and managers.

Potential participants who expressed interest received information about the study and the project. They had to meet the following inclusion criteria: (1) age ≥ 18 years; (2) residence in Germany and proficiency in German language (i.e., interviews were conducted in German); (3) belonging to one of the three following stakeholder groups: Patients (group 1, e.g., recent experience with healthcare, or suffering from chronic disease with needs for repeated care encounters, or engagement in patient self-help groups); clinical professionals and patient advocates (group 2, e.g., doctors, nursing staff, administrative personnel, patient representatives or patient advocates); and healthcare managers (group 3, e.g., in institutional risk management, health insurances). After returning the signed informed consent, participants were included into the panel.

### Data collection procedure and measures

Our data collection procedure adhered to the recommendations and criteria used for conducting Delphi studies [25]. The Delphi process comprised two rounds of data collection (06–11/2023).

In the first round, qualitative, semi-structured interviews were conducted by one study team member via the ZoomX video-conference system (Zoom Video Communications, Inc., San José, USA; Deutsche Telekom, Bonn, DE) and were audio taped. The recordings were later transcribed verbatim for further analysis according to predetermined transcription rules [26]. For the purpose of this study we developed open-ended questions that allowed for the exploration of experiences and motives regarding PE. First, the interviewer introduced shortly the focal topics of the interview, i.e., patient engagement, patient safety, healthcare. Using open-ended questions, participants were then asked concerning their interests and/or their engagement in patient safety or quality improvement measures in healthcare (exemplary questions: ‘What motivates you to take part in patient engagement activities?’, ‘what motivates patients to engage in patient safety improvement measures?’). Follow-up questions allowed participants to elaborate on their prior answers. The interview parts specifically capturing motives for PE in patient safety lasted around 10–15 min. Overall, interviews ended after all questions had been answered or after a maximum of 60 min.

The second Delphi round took place two months later, where the results of the first round (i.e., key statements) were fed back to the participants via an online survey on the unipark.de platform (Tivian XI GmbH, Cologne, DE). Panelists evaluated importance of identified motives on a Likert-scale from 1 (‘not important’) to 7 (‘very important’). To capture additional motives not being identified in round 1, an open question was additionally presented (i.e., ‘What motivates you personally to support, take part in, or implement PE in patient safety?’).

Before each Delphi-round, a cognitive pretest was conducted to test for comprehensibility and clarity. Additionally, interim results of each round were discussed within the study team.

### Analyses

We used a consecutive, step-wise analysis approach of qualitative statements, quantitative evaluations, and mapping of stakeholder motives. We first applied a qualitative content analysis approach based on Kuckartz and Rädiker [27] using MAXQDA (VERBI Software GmbH, Berlin, DE) for analyzing the interview transcripts and developing categories for reported content. Main categories were developed deductively from the interview guide, while the subcategories were developed inductively. Drawing upon the first round of data analyses and an iterative discussion among coders and within the study team, subcategories were developed and refined. Next, inter-rater agreement among two trained coders was determined in two transcripts: using MAXQDA, we obtained sufficient inter-rater reliability of Kappa_n_ = 0.76 [27, 28] across the sub categories (i.e., distinct motives of PE in patient safety). To further improve consistency, coding rules and category system were discussed again, refined, and applied to a third transcript: where a Kappa_n_ = 0.84 was achieved. The two coders then coded the remaining 31 interviews separately using the final category system. For the second round of the Delphi survey, categories were paraphrased into key statements (with iterative refinements) and key statements were attributed to stakeholder groups, respectively. Descriptive statistics of importance ratings of PE motives (mean, standard deviation) were calculated overall as well as per stakeholder group. Additionally, consensus on the importance was checked for all identified motives across stakeholder groups. Applying the approach from Anderson, Baker [22], consensus was achieved if rated as 6 (‘important’) or 7 (‘very important’) by at least 80% of panelists. We used SPSS Statistics 29 (IBM, Armonk, US).

## Results

In total, 34 participants (23 women) were recruited (average age of 56.9 years, standard deviation: 10.8). In terms of the three stakeholder groups, the sample was composed of 16 patients, nine healthcare professionals (including 3 clinicians) as well as nine managers from healthcare institutions.

In the first Delphi-round, statements on PE motives were identified in 29 interviews. In the remaining five interviews, participants did not report any specific motives. Of the 34 study participants in the first round, 33 also took part in the follow-up online survey (response rate 97%). Of the 33 respondents in the second round, 29 also filled in the additional open question regarding their personal motives.

### Identification of stakeholder motives for patient engagement in patient safety

**Fig. 1:**
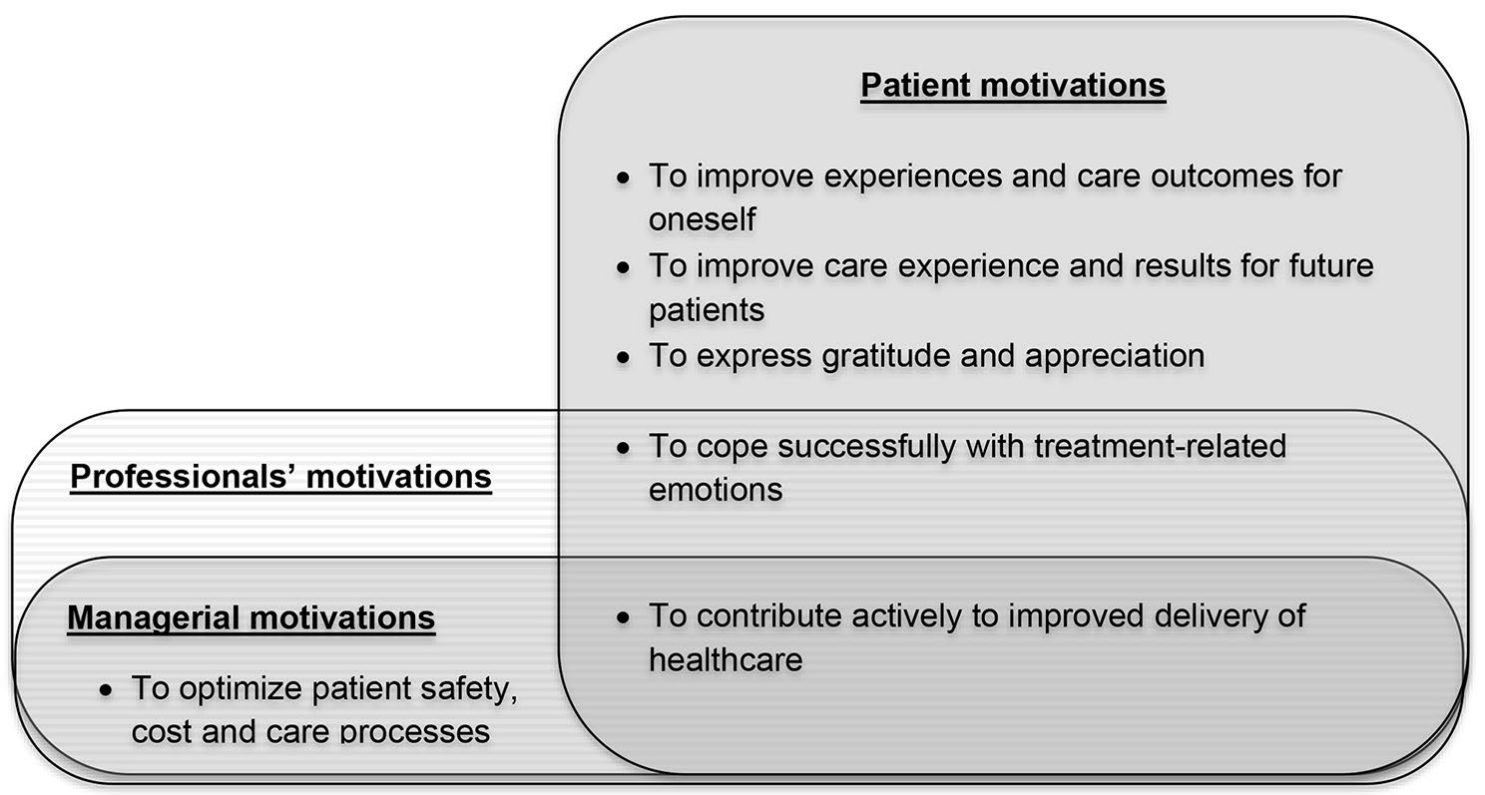
Concept map of elicited stakeholder motives for patient engagement in patient safety

We obtained *n* = 91 individual statements. In course of the analyses, seven qualitatively different motives emerged, ranging from 4 to 22 interview statements per motive. Together with open text replies of the second Delphi-round, we eventually identified the following distinct and shared motives among three stakeholder groups (cf., Fig. 1):

#### Description of stakeholder motives

For the group of patients, several motives were identified with the following exemplary statements: First, motivation for an *improved care experience and results for future patients* (e.g. interviewee #6: ‘They [patients] don’t want anyone else to suffer the same harm. They actually want a clarification or a conversation with the person in charge.’). A second motive was *expressing gratitude and appreciation* towards the care institution and past treatment (e.g. interviewee #4: ‘I think […] that would be a clinic that would deserve a good rating.’). Another motive underlined patients’ need for *coping successfully with treatment-related emotions*, and particularly emphasizing self-efficacy (e.g. interviewee #31: ‘the moment I can do something myself, I lose my helplessness. And I think that’s an incredibly important point.’). To this end, overcoming experiences of not being heard or taking (back) an active role also were mentioned (e.g. interviewee #31: ‘I know from my own experience how much safety it gives me when the doctors talk to me – not about me.’). Further motive was *improvement of experience and care outcomes for oneself*, referring to personal benefits (e.g. interviewee #33: ‘Yes, I would also like to solve the problem for others, but first I want to solve it for myself’). This included better coping with illness and recovery (e.g. interviewee #11: ‘If you feel safe and well looked after as a patient, this also promotes the recovery process or ability to live well with an illness.’).

The *active contribution to improved delivery of healthcare* was identified as a joint motive shared among patients and care professionals. This encompassed statements such as considering patients’ unique perspectives (e.g., participation in patient advisory boards), drawing on their own personal experiences, and providing opportunities to actively engage (e.g. interviewee #28: ‘Patients and their relatives are often the only ones who know the entire course of the illness or treatment. Giving them a voice is very important.’).

Among professionals and institutional representatives (i.e. managers), a frequently shared motive was *optimizing patient safety*,* costs*,* and care processes* that combined different topics. It encompassed various statements, including detection of errors, improvement of care quality, closing gaps in care, ensuring higher efficiency in patient safety improvements, and achieving cost savings (e.g. interviewee #6: ‘I want to achieve an improvement in quality.’).

We were able to identify one motive shared among all stakeholders: this was a collective aspiration toward the *improvement of patient-provider relationships.* Herein, we captured statements on fostering mutual trust, promoting effective and non-hierarchical communication as well as respecting patient experiences after incidents. A key statement was: ‘…the way we communicate, taking seriously what patients and relatives report back to us. The effect on patients should not be underestimated.’ (interviewee #20).

Beyond the seven key motives, we further captured a spectrum of statements that were unspecific and were not attributed to one of the major motive categories. Herein, further motives referred to volunteering driven by a sense of personal meaning and a desire for social contribution, joy of seeing successes of individual efforts, as well as discontents with the existing status quo. Since these motives were only sparsely mentioned and for the sake of survey brevity, we did not transfer these motives into round 2.

### Importance ratings of motives for patient engagement in patient safety

In the second round, all panelists rated identified motives. The results are presented in Table 1. Although we observed slightly higher scores among the patient group, we did not obtain significant mean differences across stakeholder groups (results not reported here).

We further determined consensus among panelists (cf., Table 1). Agreement was obtained for four motives: *to improve experiences and care outcomes for oneself; to improve care experience and results for future patients; to improve patient-provider relationships; to optimize patient safety*,* costs*,* and care processes*. In an additional, exploratory step, we determined consensus solely within the patient group. Here we found that two other motives received high approval: *to express gratitude and appreciation* (with 100%) and *to contribute actively to improved delivery of healthcare* (86.7%).

**Table 1:** Panellists’ ratings of motive for PE in patient safety (overall and per stakeholder group)

Motives	Overall	Stakeholder group			Consensus overall
		Patients	Professionals	Managers	
	M (SD)	M (SD)	M (SD)	M (SD)	(in %)
To improve experiences and care outcomes for oneself	6.55 (1.09)	6.69 (0.79)	6.50 (1.41)	6.33 (1.32)	**87.9**
To improve care experience and results for future patients	6.48 (1.03)	6.56 (1.03)	6.25 (1.17)	6.56 (1.01)	**87.9**
To improve patient-provider relationships	6.44 (1.05)	6.53 (0.92)	6.50 (0.93)	6.22 (1.39)	**81.2**
To optimize patient safety, costs, and care processes	6.31 (0.82)	6.60 (0.63)	6.25 (0.71)	5.89 (1.05)	**84.4**
To contribute actively to improved delivery of healthcare	6.22 (1.31)	6.40 (1.45)	5.88 (1.13)	6.22 (1.30)	78.1
To express gratitude and appreciation	5.81 (1.51)	6.73 (0.46)	4.63 (1.92)	5.33 (1.41)	68.7
To cope successfully with treatment-related emotions	5.76 (1.32)	5.94 (1.24)	5.50 (1.70)	5.67 (1.23)	57.6

Notes: Overall group (*n* = 33), stakeholder groups: Patients (*n* = 16), healthcare professionals (*n* = 8), managers, institutional representatives (*n* = 9). M Mean, SD Standard deviation. Scale range: 1 (‘not important’) to 7 (‘very important’), Consensus defined as agreement on scale levels 6 and 7. Bold: consensus > 80%

## Discussion

Despite frequent calls for extended engagement of patients in patient safety, respective efforts are often difficult and lack sustainable success. Previous research often looked at the benefits of engaging patients in patient safety as well as relevant facilitators to the process [6, 9, 29, 30]. Yet, underlying motives of both patients and further key stakeholders for engaging in such patient safety enhancements have not yet been thoroughly and comprehensively investigated empirically [16]. Since divergence of attitudes and motives may hinder ample inclusion of all relevant stakeholders, our findings may help to identify overarching motives that facilitate in engaging stakeholders in collaborative patient safety practices [31].

In the light of these shortcomings and to overcome common obstacles in design and implementation, a deeper understanding of specific motives for engaging in patient safety is essential. Drawing upon a multi-stakeholder perspective, this investigation identified stakeholder motives that could be taken into account in future adoption of patient engagement in patient safety. Our findings may thus inform future improvement efforts and contribute to the co-design and implementation PE initiatives, e.g., that in early stages of implementation, attitudes and willingness of all relevant stakeholders are assessed, jointly reflected upon, and considered during ensuing steps [32]. Specifically, we assume that the insights of this multi-perspective, multi-methods, and stepwise investigation contribute particularly in the following manner to the current evidence base.

Our interview data revealed a range of motives among relevant stakeholder groups in patient safety. Firstly, the results suggest that different motives for engaging in patient safety come into play and need to be considered. Nonetheless, some motives have been mentioned repeatedly such as improvement of the patient-provider relationship as well as overall improvement and optimization of patient safety, costs and processes in care. A recent review just emphasized that facilitating circumstances in successful patient engagement in patient safety practices also include perceived value of patient-professional collaboration [32]. Our findings also concur well with previous reports on institutional motives of improving the quality of care [19], the efficiency of healthcare systems [20] and lowering of healthcare costs [18]. We also found in the interviewees’ responses, that within patients’ and providers’ statements, concepts of safety, poor care delivery, and increased costs were overlapping and interlinked. Both patients and healthcare professionals expressed a desire to actively contribute their own experiences to improve healthcare, even when these experiences are not directly related to specific safety incidents. Many patients wish to share their personal experiences to create meaningful changes in healthcare and to expand their often limited influence on the system [15, 16]. Additionally, valuable feedback can improve the work life of clinicians and staff [18]. Lastly, patient involvement fuels experiences of self-fulfillment [16]. Nonetheless, the variety of motives attributed to patients is manifold and may include further motives that have not been fully captured within our investigation, e.g., learning new insights, obtaining compensation or other perks [16].

We also found that different stakeholders share similar motives for PE in patient safety. As reported above, we identified shared motives that were previously attributed primarily to one specific stakeholder group [16]. We observed that shared motives included *optimization of patient safety*,* costs and care processes* and *improvement of the patient-provider relationships* as well as *contributing actively to improved delivery of healthcare*. This finding provides empirically-based insights and expands previous research that almost exclusively focused on patients’ perspectives, yet, acknowledging that further stakeholders and parties might bear different as well as shared motives [16]. Nonetheless, our approach to determine shared motives warrants careful interpretation and future refinement. Post-hoc, we cannot infer if different motives share common meaning and semantics, what may have resulted in high consensus ratings. For example, the motive *to optimize patient safety*,* costs and care processes* might be conceived semantically similar as *to improve care experience for future patients/themselves*. Previous research has shown that patients’ conceptualizations of safety, responsibility, and their engagement in safety work is constructed differently and affected by context [33]. Moreover, we did not obtain consensus on the motive *to contribute actively to improved delivery of healthcare*. Post-hoc, we assume that patients and providers may diverge in perceived agency and control when it comes to actual changes or challenging behaviors, especially in healthcare environments with low psychological safety [31, 34]. Consideration of patients’ as well as providers’ motives may help to inform the co-design and implementation of safety improvement with greater relevance to patients and legitimacy to clinicians [33].

To this end, our exploratory results are preliminary, and our novel findings thus call for future research into common expectations as well as tailored communication approaches when it comes to addressing several stakeholder groups for joint collaboration in the course of patient engagement in patient safety improvement.

### Strengths and limitations

Our investigation followed an exploratory approach, applying a two-step Delphi procedure, to convey relevant stakeholder motives for PE in patient safety. A potential deficit of Delphi methods of neglecting extreme and unconventional opinions by forming a consensus does not apply to this study, as the qualitative results were reported separately in the first step. Another weakness of the Delphi method is that expertise is unevenly spread across participants [35]. However, reporting on personal motives did not require any deeper expertise from the participants. A major advantage of the method is, that it can be tailored to the research topic, especially when knowledge is incomplete and the objective is to improve a joint understanding [36]. Furthermore, we recruited a heterogeneous panel representing various stakeholders of the German healthcare system. Additionally, we achieved a high response rate and high consensus in various motive categories, what supports the validity of our consolidated statements [22].

Nonetheless, our results should be interpreted in the light of some major and minor limitations. Our data was based on subjective reports across several stakeholders in Germany what may limit external validity. A priori, we did not specify or limit statements to specific patient safety activities, challenges, and domains. We rather pursued a generic approach without tailoring it to one particular topic. Future investigations should seek to replicate our findings across different healthcare sectors, national settings, stakeholder groups, and domains of patient safety activities, e.g., post-incident analyses, patient advisory boards, representative roles in institutional activities to reduce patient harm. Moreover, future research should strive for in-depth analyses of motives for PE in and outside of the domain of patient safety. Although our investigation focused specifically on PE in the area of patient safety, we acknowledge that various motives of PE are fundamental to healthcare improvement and are relevant to other domains such as quality improvement, patient-centered care, or organizational development [33, 37]. We assume that similar motives may play a key role, such as feelings that engagement is worthwhile, appreciated, and positively rewarded [38]. Nonetheless, successful patient engagement in safety improvement depends upon multiple system-factors such as perceived confidence, non-paternalistic culture, psychological safety, and wider organizational resources, e.g., financial reimbursement [38]. The majority of our panelists reflected on hospital based events and interventions. Future surveys should attempt to convey motives for PE in outpatient care. Our assignment of motives to the three stakeholder groups was based on panelists’ attributions and suggestions what needs to be confirmed in future research. Our mapping of stakeholder motives should therefore be considered as a base for quantitatively focused evaluation methods in the future. Further limitations refer to the representativeness of our sample, the limited sample size, and selection bias of participants. Our three stakeholder groups had unequal sizes, what may have introduced bias. However, since we finally obtained mean ratings and consensus metrics, potential bias may be limited. Moreover, since we tested for discrepancies between stakeholder groups, we assume that major differences would have been identified. We may have excluded important stakeholders (i.e., regulators, funding bodies). Our group difference tests were computed without prior determination for statistical power. Our pragmatic approach of clustering our interviewees needs to be taken into account, since we collated clinicians and patient advocates into one stakeholder group (mainly because of the small number of individuals per role as well as their perspective into patient safety across various institutions and settings). Lastly, as often in PE practice in real-world healthcare, roles may overlap. Specifically, institutional representatives (i.e., managers) as well as professionals are also patients themselves (or, vice versa, patient advocates may have a healthcare background), what eventually may also account for the overlap of motives across stakeholder groups, similarly to the recent Canadian study where a third of the patient and family participants also showed a healthcare background [16].

### Implications for healthcare practice and future research

Motivation is a major factor in determining whether intended actions are successfully carried out as planned. Therefore, our reported motives could inform future approaches to address key stakeholder motives in patient safety practices that capitalize on effective and sustainable PE. For example, for co-design and implementation of PE in patient safety practices, recurrent consideration of motives across stakeholders may help to avoid divergence and align attitudes, what ultimately fosters processes of involvement. Our results corroborate the multifaceted nature of expectations and priorities towards PE in patient safety [31]. Notwithstanding this broad spectrum, high convergence across stakeholder groups promotes joint social identities that supports implementation of patient safety practices [21]. Establishing common motives could be utilized to foster a sense of belonging between stakeholders and ultimately stipulate the attainment of collective goals, to eventually unleash the full potential of PE interventions. However, different attitudes and motives also emphasize the need to provide a range of equally valued opportunities for patients to be involved at different levels and in differing roles [5, 31].

Our findings might be conceived as a base for further exploration of underlying motivational processes in PE and for development of systemic approaches for effective PE in patient safety that successfully address attitudes and concerns of different relevant stakeholders. Moreover, future systematic literature assessments may serve as a foundation for subsequent, comprehensive expert surveys to consolidate their opinions concerning motives across a wider thematic spectrum that draws upon multiple publications (i.e., across different settings, sectors, and national contexts). Furthermore, the motives acquired in our study could be operationalized for evaluation and validation purposes (e.g., a user-friendly survey tool to capture PE motives). Finally, we assume that motives for PE may change substantially in the course of involvement and implementation processes. Development of respective frameworks that elicit stakeholder motives across time and stages of extended durations of involvement is thus necessary, i.e., to determine temporal influences which may feedback on stakeholder motives. Such a framework could subsequently be tested empirically and used for efficient implementation and evaluation of future PE interventions aiming at patient safety.

## Conclusion

Despite the growing interest for PE in patient safety, underlying stakeholder motives have been rarely investigated. Our exploratory analysis across key stakeholder groups including patients, clinicians, and managements revealed a wide range of distinct as well as shared motives. Our consolidated findings inform future PE interventions and collaborative efforts aimed at improving safe delivery of healthcare for all stakeholders.

## Data Availability

The anonymized raw datasets of statements (in German language) and motivation categories that were used during the current study are available from the corresponding author on reasonable request.
